# Identification of a Hypoxia-Related Molecular Classification and Hypoxic Tumor Microenvironment Signature for Predicting the Prognosis of Patients with Triple-Negative Breast Cancer

**DOI:** 10.3389/fonc.2021.700062

**Published:** 2021-08-19

**Authors:** Xiaoli Sun, Huan Luo, Chenbo Han, Yu Zhang, Cunli Yan

**Affiliations:** ^1^Department of Medical Oncology, Baoji Maternal and Child Health Hospital, Baoji, China; ^2^Department of Breast Surgery, Baoji Maternal and Child Health Hospital, Baoji, China; ^3^Department of General Surgery, Baoji Maternal and Child Health Hospital, Baoji, China

**Keywords:** triple-negative breast cancer, hypoxic tumor microenvironment, prognostic model, qRT-PCR, molecular classification

## Abstract

**Purpose:**

The hypoxic tumor microenvironment was reported to be involved in different tumorigenesis mechanisms of triple-negative breast cancer (TNBC), such as invasion, immune evasion, chemoresistance, and metastasis. However, a systematic analysis of the prognostic prediction models based on multiple hypoxia-related genes (HRGs) has not been established in TNBC before in the literature. We aimed to develop and verify a hypoxia gene signature for prognostic prediction in TNBC patients.

**Methods:**

The RNA sequencing profiles and clinical data of TNBC patients were generated from the TCGA, GSE103091, and METABRIC databases. The TNBC-specific differential HRGs (dHRGs) were obtained from differential expression analysis of hypoxia cultured TNBC cell lines compared with normoxic cell lines from the GEO database. Non-negative matrix factorization (NMF) method was then performed on the TNBC patients using the dHRGs to explore a novel molecular classification on the basis of the dHRG expression patterns. Prognosis-associated dHRGs were identified by univariate and multivariate Cox regression analysis to establish the prognostic risk score model.

**Results:**

Based on the expressions of 205 dHRGs, all the patients in the TCGA training cohort were categorized into two subgroups, and the patients in Cluster 1 demonstrated worse OS than those in Cluster 2, which was validated in two independent cohorts. Additionally, the effects of somatic copy number variation (SCNV), somatic single nucleotide variation (SSNV), and methylation level on the expressions of dHRGs were also analyzed. Then, we performed Cox regression analyses to construct an HRG-based risk score model (3-gene dHRG signature), which could reliably discriminate the overall survival (OS) of high-risk and low-risk patients in TCGA, GSE103091, METABRIC, and BMCHH (qRT-PCR) cohorts.

**Conclusions:**

In this study, a robust predictive signature was developed for patients with TNBC, indicating that the 3-gene dHRG model might serve as a potential prognostic biomarker for TNBC.

## Introduction

Breast cancer is reported to be one of the most common causes of cancer-related deaths among females around the world, which is a heterogeneous tumor, resulting in variable clinical features (1). Triple-negative breast cancer (TNBC), characterized by high histological grade and high rates of metastasis, is the most aggressive subtype of breast cancer (2). TNBC lacks the expression of estrogen receptor (ER), progesterone receptor (PR), and human epidermal growth factor receptor 2 (HER2) and constitutes 12%–18% of breast cancer patients (3). TNBC patients are not eligible for endocrine therapy or anti-Her2 therapy and with poor survival (2, 4). Hence, exploration of new therapeutic target and investigations of clinically applicable predictors are indispensable for TNBC.

The hypoxia-related mechanism has long been considered as one of the hallmarks in the cancer signaling pathway (5–7). Hypoxic tumor microenvironment (TME) has been reported to modulate each step in the metastatic process (8) and regulate multiple cancer phenotypes (9). Targeting hypoxia will thus inhibit several traits of tumor progression, metastasis, radioresistance, and chemoresistance (10, 11), which has been an important focus of TNBC therapy. According to a previous study, certain hypoxia-related genes (HRGs) and their mediators, hypoxia-inducible factors (HIFs), may serve as prognostic predictors and therapeutic targets in breast cancer (9). However, a systematic analysis of the prognostic prediction models based on multiple HRGs have not been established in TNBC before in the literature.

In the present study, we firstly investigated the differentially expressed genes (DEGs) by using the differential expression of MDA-MB-143 cell lines under normoxia and hypoxia conditions in the public database. Then, we took the genes shared by HRGs, which was obtained from Gene Ontology (GO), Kyoto Encyclopedia of Genes and Genomes (KEGG), and Reactome databases, and DEGs as dHRGs (TNBC-specific hypoxia-related genes) for follow-up research. Multiple TNBC datasets with clinical information, such as The Cancer Genome Atlas (TCGA), Gene Expression Omnibus (GEO), and cbioportal, were utilized to study the relationship between the prognosis of TNBC patients and the expression patterns of dHRGs, and a prognostic nomogram was further confirmed and validated in TCGA, GSE103091, and METABRIC databases. Finally, we analyzed the effects of factors such as somatic copy number variation (SCNV), somatic single nucleotide variation (SSNV), and methylation level on the expressions of dHRGs.

## Materials And Methods

### Data Acquisition and Processing

The gene expression profiles of the GSE104193 and GSE33950 datasets were downloaded from the GEO database (http://www.ncbi.nlm.nih.gov/geo/), where the MDA-MB-231 cell lines were cultured under normoxic (20%–21% oxygen) and hypoxic (1%–1.5% oxygen) conditions. We obtained four normoxia and four hypoxia cultured tumor cells from GSE104193 using Illumina HiSeq 2000, and enrolled four normoxia and four hypoxia cultured tumor cells from GSE33950 using Affymetrix Human Genome U133 Plus 2.0 Array [HG-U133_Plus_2] (**Table 1**). The level three RNA sequencing data and clinicopathological information of 1,223 breast patients were downloaded from TCGA (http://portal.gdc.cancer.gov/), 238 samples from GSE103091 (Affymetrix Human Genome U133 Plus 2.0 Array [HG-U133_Plus_2]), and 2,509 samples from METABRIC (http://www.METABRIC.org/) datasets. Only TNBC patients with survival time and status remained. The gene expression profiles of TNBC patients were finally composed of 115 TCGA samples, 107 GSE103901 samples, and 298 METABRIC samples. The demographics and clinical features of the TNBC patients in the TCGA, GSE103091, and METABRIC cohort are displayed in **Table 2**.

**Table 1:** Information of data for differential expression analysis of normoxia and hypoxia cultured cells.

Data	Sample ID	Source	Treatment	Type
GSE104193	GSM2791577	cell line: MDA-MB-231	Hypoxia	rep1
	GSM2791581	cell line: MDA-MB-231	Hypoxia	rep2
	GSM2791585	cell line: MDA-MB-231	Hypoxia	rep3
	GSM2791589	cell line: MDA-MB-231	Hypoxia	rep4
	GSM2791576	cell line: MDA-MB-231	Normoxia	rep1
	GSM2791580	cell line: MDA-MB-231	Normoxia	rep2
	GSM2791584	cell line: MDA-MB-231	Normoxia	rep3
	GSM2791588	cell line: MDA-MB-231	Normoxia	rep4
GSE33950	GSM839357	cell line: MDA-MB-231	Hypoxia	biological replicate A
	GSM839358	cell line: MDA-MB-231	Hypoxia	biological replicate B
	GSM839359	cell line: MDA-MB-231	Hypoxia	biological replicate C
	GSM839360	cell line: MDA-MB-231	Hypoxia	biological replicate D
	GSM839353	cell line: MDA-MB-231	Normoxia	biological replicate A
	GSM839354	cell line: MDA-MB-231	Normoxia	biological replicate B
	GSM839355	cell line: MDA-MB-231	Normoxia	biological replicate C
	GSM839356	cell line: MDA-MB-231	Normoxia	biological replicate D

**Table 2:** Demographics and clinicopathological features of patients in the TCGA, GSE103091, METABRIC, and BMCHH cohort.

Clinical Features	TCGA(*n* = 115)	GSE103091(*n* = 107)	METABRIC(*n* = 298)	BMCHH(*n* = 52)
**OS**				
Alive	97	78	137	42
Dead	18	29	161	10
**T Stage**				
T1	26			6
T2	73			41
T3	12			3
T4	4			2
**N Stage**				
N0	74			37
N1	25			9
N2	12			4
N3	4			2
**M Stage**				
M0	97			51
M1	2			1
Mx	16			0
**Stage**				
I	19		62	5
II	72		130	39
III	19		25	7
IV	2		0	1
X	3		81	0
**Grade**				
G1			3	
G2			36	
G3			257	
GX			2	
**Age**				
≤55	65	47	142	35
>55	50	60	156	17

### Identification of TNBC-Specific HRGs

As reported by Wang et al. (12), a total of 1,694 genes in 65 gene sets were selected as HRGs with the following keywords: hypoxia AND Homo sapiens (**Supplementary Table S1**). All the known HRGs were screened from GO, KEGG, and Reactome databases by using the Molecular Signatures Database (MSigDB), a collection of annotated gene sets. Differential expression analysis between normoxic and hypoxic culture was screened in both GSE104193 and GSE33950 cohorts. The GSE104193 cohort was Counts data using RNA-Seq and was analyzed using the DESeq2 package in R. The GSE33950 cohort was chip data, which was analyzed using limma package in R. |Fold change (FC)| > 1.5 and FDR < 0.05 were considered as the cutoff criteria for determining DEGs. The dysregulated genes of GSE104193 and GSE33950 in hypoxia were visualized by volcano plots. Finally, the genes in the intersection of the HRGs and the DEGs of GSE104193 and GSE33950 were considered as TNBC-specific HRGs (defined as differential HRGs, dHRGs) for further analysis and were displayed by a Venn diagram.

### Molecular Classification and Prognostic Analysis of TNBC

The association between the dHRG expressions and patients’ overall survival (OS) was evaluated by the univariate Cox regression analysis in the TCGA-TNBC and GSE103091 cohort by using coxph function of the survival package in R. The prognosis-related genes (PRGs) with *p*-value < 0.05 in the TCGA-TNBC and GSE103091 cohort were merged. By performing non-negative matrix factorization (NMF) with the “brunet” method for 50 iterations, we clustered the TCGA-TNBC and GSE103091 cohort. The clustering number *k* was set as 2 to 10, and we further determined the average profile width of common member matrix by using NMF package in R with the minimum member numbers of each subclass set to 10. According to indexes including cophenetic, dispersion, and silhouette, the optimal number of clusters was finally determined. Kaplan–Meier (K–M) survival curves were analyzed to show the difference in survival rates between different groups.

### Methylation Analysis of HRGs

Spearman rank correlation coefficient between TCGA-specific dHRGs and methylation sites was calculated using the “cor.test” function in R, and methylation sites in TSS200, TSS1500, and gene body areas with coefficient > 0.6 and *p*-value < 0.05 were selected. M6A-related genes were derived from reference (13), and 17 critical genes in the m6A process (writers, erasers, and readers) were sorted out. The correlation coefficient between expression level and methylation level of the dHRGs and expression level of the m6A related gene was then evaluated.

### SCNV and SSNV Analysis of HRGs

Firstly, CNV intervals of TCGA-TNBC CNV data were arranged using the following criteria (1): intervals with more than 50% overlap were merged (2); intervals with less than five overlay probes were removed (3); CNV intervals were mapped to the corresponding genes based on “gencode.v32” (version GRh38) (4); CNV regions belonging to the same gene region were merged, and the merged CNV values were averaged. Spearman rank correlation coefficient between the expression levels and CNV levels of HRGs were assessed using the “cor.test” function in R, with *p*-value < 0.05. For the SNV data, the mutect2 version of TCGA SSNV data was obtained, and then the intron interval and the mutations annotated as silence were removed.

### Construction of the HRG-Based Prognostic Model

Based on the prognostic dHRGs obtained from the univariate Cox regression analysis, the prognostic risk score model was constructed by the following multivariate Cox analysis. Then, the risk score was calculated for each patient. All TNBC patients were categorized into high-risk and low-risk groups by using the median value of their risk score. K–M survival curve was constructed to estimate the survival differences of patients with high or low risk scores. The prognostic performance was evaluated by the time-dependent receiver operating characteristic (ROC) curve analysis within 1, 3, and 5 years to evaluate the predictive accuracy of the prognostic model by using the survcomp and survivalROC package in R.

### Quantitative Real-Time Polymerase Chain Reaction (qRT-PCR)

We collected 52 freshly frozen TNBC and 52 paired adjacent normal samples from Baoji Maternal and Child Health Hospital (BMCHH) between January 2017 and January 2018. All specimens were confirmed by the pathological diagnoses. This study was approved by the Research Ethics Committee of BMCHH. Written informed consents were obtained from all participants included in the study. The primers used for qRT-PCR are shown in **Supplementary Table S2**. Total RNA was isolated from samples using the Trizol reagent (Thermofisher, Cat. No. 15596026). The cDNA was synthesized by using cDNA reverse transcription kit (TOYOBO, Cat. No. FSQ-101), and the resulting cDNA was amplified by the SYBR Green PCR kit (Applied Biosystems, Cat. No. 4368708). Samples were tested by the QuantStudio 5 Real-Time PCR System (Applied Biosystems, Thermo Fisher Scientific) according to the manufacturer’s instructions. Each experiment was performed at least three times. The expressions of the target genes were calculated using the 2^−ΔΔCt^ method relative to the control housekeeping gene GAPDH.

### Validation of the 3-Gene dHRG Signature in the BMCHH Cohort

The clinical and follow-up data of the BMCHH validation cohort are shown in **Table 2**. According to the formula of the 3-gene dHRG signature constructed before, the risk score was generated for those patients in the BMCHH cohort. Then, they were divided into high- and low-risk groups by using the median value of their risk score. K–M survival curve was also performed to estimate the survival differences of high- and low-risk patients. In addition, we also performed time-dependent ROC analysis to assess the prognostic performance of the 3-gene dHRG signature within 1, 3, and 5 years.

## Results

### Identification of TNBC-Specific HRGs and Enrichment Analyses

**Figure 1:**
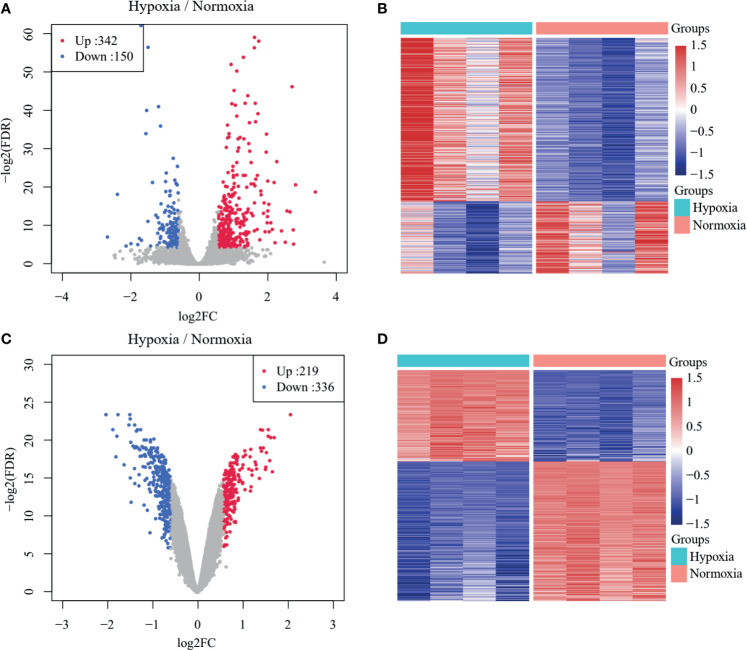
Identification of TNBC-specific hypoxia-related genes (HRGs). **(A)** Volcano plot of differentially expressed genes (DEGs) between normoxic and hypoxic cultured breast cancer cells in GSE104193. **(B)** Heat map of DEGs between normoxic and hypoxic cultured breast cancer cells in GSE104193. **(C)** Volcano plot of DEGs between normoxic and hypoxic cultured breast cancer cells in GSE33950. **(D)** Heat map of DEGs between normoxic and hypoxic cultured breast cancer cells in GSE33950.

The overall workflow of the present study is presented in **Supplementary Figure S1**. A total of 492 DEGs in GSE104193 were screened between normoxia and hypoxia cultured breast cancer cells, including 242 upregulated genes and 150 downregulated genes (**Supplementary Table S3**), and were displayed in volcano plots and heat maps (**Figures 1A, B**). A total of 555 DEGs in GSE33950 were identified in GSE33950, namely, 219 upregulated genes and 336 downregulated genes (**Figures 1C, D** and **Supplementary Table S3**).

**Figure 2:**
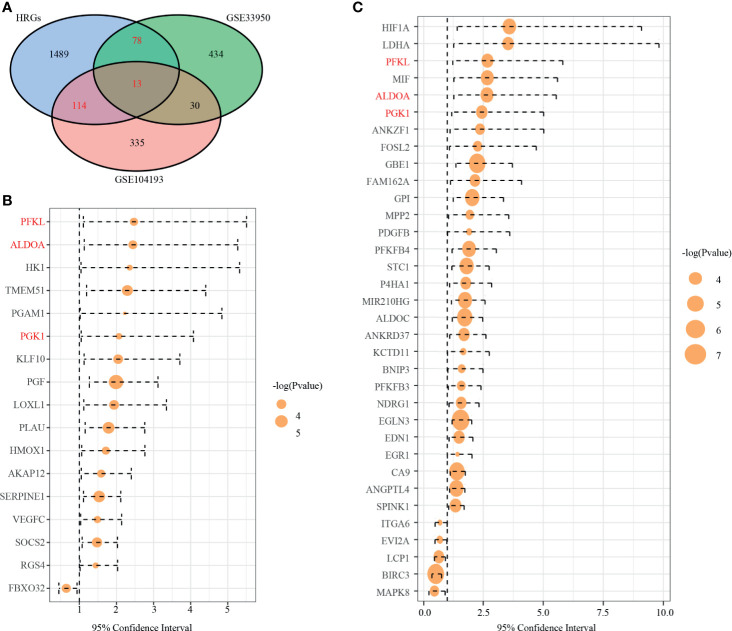
Identification of prognostic TNBC-specific hypoxia-related genes (HRGs). **(A)** Venn diagram of the 13 TNBC-specific HRGs, which are the genes in the intersection of the HRGs from MSigDB and the DEGs of GSE13041 and GSE33950. **(B)** Hazard ratio (HR) of univariate survival analysis of HRGs in TCGA-TNBC. **(C)** HR of univariate survival analysis of HRGs in GSE13041. (Genes with *p*-value < 0.05 are shown in **B**, **C**).

We drew a Venn diagram, including DEGs from GSE104193 and GSE33950 and HRGs (**Figure 2A**). There were 127 shared genes between the HRGs and DEGs from GSE104193, and 91 shared genes between the HRGs and DEGs from GSE33950. A total of 205 shared genes among DEGs from GSE104193 and GSE33950 and HRGs were found, which is called dHRGs in the following.

**Figure 3:**
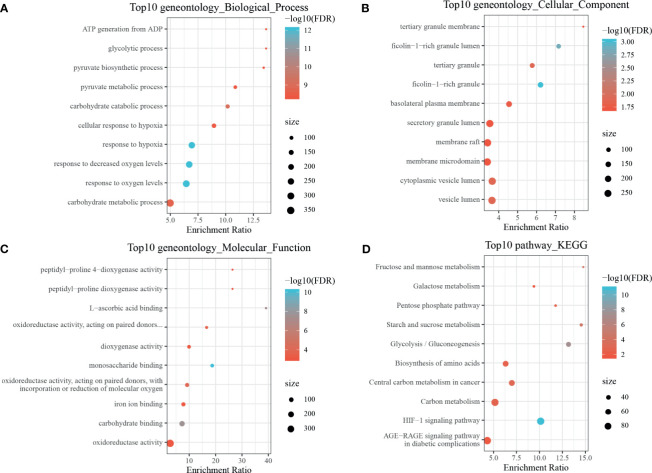
Enrichment analyses of TNBC-specific dHRGs. Biological processes **(A)**, cellular components **(B)**, molecular functions **(C)**, and Kyoto Encyclopedia of Genes and Genomes (KEGG) pathways **(D)** enriched in the TNBC-specific HRGs.

By using the WebGestaltR (v0.4.2) R package, enrichment analysis was performed on dHRGs to investigate the molecular mechanisms in the tumorigenesis and progression of TNBC (**Supplementary Table S4**). In the biological process (BP) category, dHRGs were significantly enriched in response to hypoxia, oxygen levels, decreased oxygen levels, and cellular response to hypoxia (**Figure 3A**). In the cellular component (CC) category, dHRGs were significantly enriched in the secretory granule lumen, membrane raft, membrane microdomain, cytoplasmic vesicle lumen, and vesicle lumen (**Figure 3B**). In the molecular function (MF) category, dHRGs were significantly enriched in the oxidoreductase activity (**Figure 3C**). KEGG pathway analysis demonstrated that dHRGs were mainly enriched in the HIF-1 signaling pathway, Carbon metabolism, and AGE-RAGE signaling pathway in diabetic complications (**Figure 3D**).

### dHRGs-Based Molecular Classification of TNBC Patients and Associations With Prognosis

To explore the relationship of dHRGs and prognosis of TNBC patients, we performed univariate Cox analysis on 205 dHRGs in the TCGA-TNBC and GSE103901, respectively. In TCGA-TNBC, 17 of 205 dHRGs were identified to be associated with prognosis, including 16 poor-prognosis factors and 1 good prognostic factor (**Figure 2B** and **Supplementary Table S5**). In GSE103901, 34 genes were associated with prognosis, of which 29 genes were poor-prognosis-related and 5 genes were good-prognosis-related (**Figure 2C** and **Supplementary Table S5**). A total of 48 genes were identified to be associated with prognosis of TNBC patients; PEKL, ALDOA, and PGK1 were poor prognostic factors in both sections.

**Figure 4:**
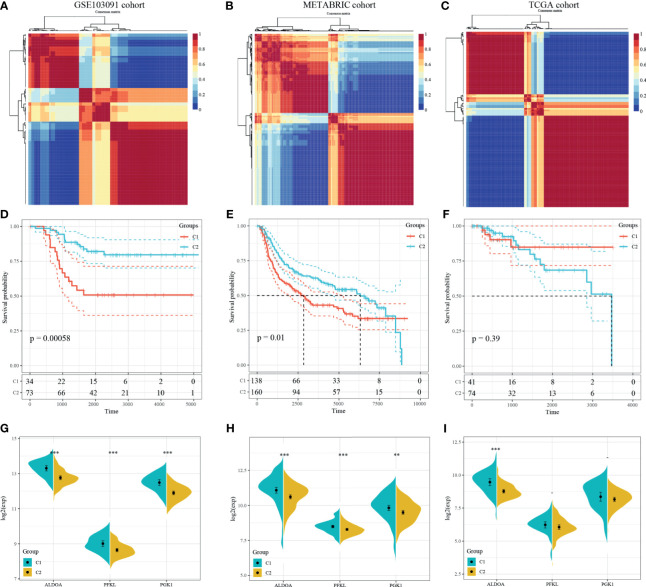
Identification and validation of an HRG-based molecular classification of TNBC patients using the nonnegative matrix factorization (NMF). Consensus clustering matrix for *k* = 2–10, which was the optimal cluster number in the GSE103901 cohort **(A)**, METABRIC cohort **(B)**, and TCGA-TNBC cohort **(C)**. Kaplan–Meier (K–M) survival analyses of the patients in Cluster 1 and Cluster 2 subgroups in the GSE103901 cohort **(D)**, METABRIC cohort **(E)**, and TCGA-TNBC **(F)**, which indicated that the patients in Cluster 1 had poorer OS than those in Cluster 2. The expression patterns of the three HRGs included in the hypoxia signature between two clusters of TNBC patients in the GSE103901 cohort **(G)**, METABRIC **(H)**, and TCGA-TNBC **(I)**. (**represents *p* < 0.01, ***represents *p* < 0.001).

NMF algorithm was then performed on the 107 GSE103901 TNBC patients using the above 48 genes, to explore a novel HRG-based molecular classification of TNBC. According to the cophenetic, dispersion, and silhouette curves, the optimal number of subgroups was determined as two (*k* = 2) (**Figure 4A**). All TNBC patients were divided into two clusters, including 34 patients in Cluster 1 and 73 patients in Cluster 2. K–M survival curve showed that Cluster 1 patients had worse OS than Cluster 2 (*p* = 5.8 × 10^-4^, **Figure 4D**). Then, we applied the same method to validate the molecular classification in METABRIC and TCGA-TNBC patients. As shown in **Figures 4B, C**, 298 METABRIC and 115 TCGA-TNBC patients were divided into two clusters, respectively. In METABRIC patients, Cluster 1 (138 patients) showed significantly worse OS than Cluster 2 (160 patients) (**Figure 4E**). However, there was no difference between the TCGA-TNBC classification groups (**Figure 4F**), which might be related to the relatively low proportion of death samples in the TCGA-TNBC data. At the same time, we compared the expression of PEKL, ALDOA, and PGK1 among different clusters of three groups. In both GSE103091 and METABRIC patients, the expression levels of the three genes in Cluster 1 was significantly higher compared with that in Cluster 2 (**Figures 4G–I**).

### Methylation Characteristics of dHRGs

**Figure 5:**
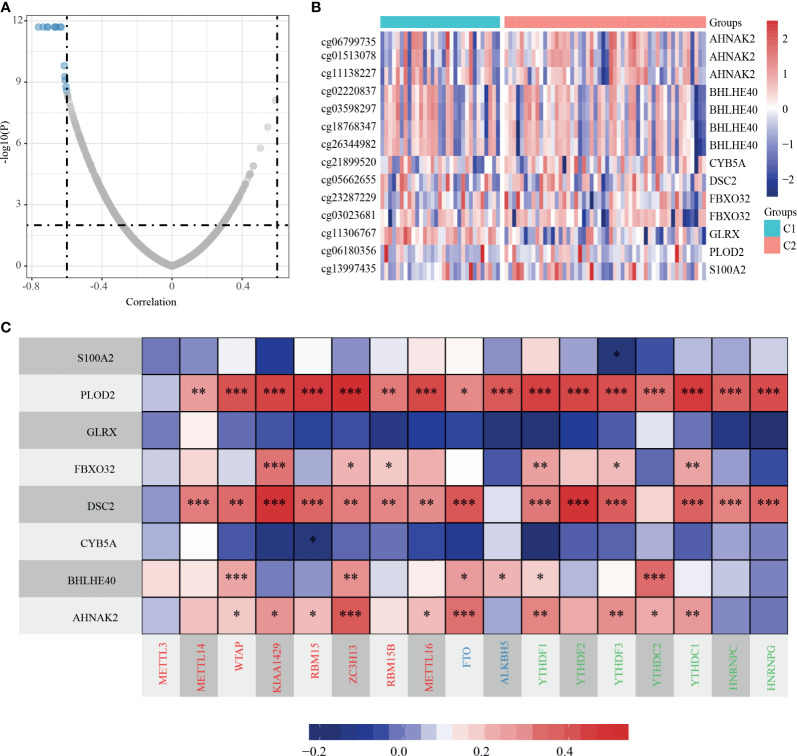
Methylation characteristics of dHRGs. **(A)** The correlation coefficient and *p*-value of dHRGs-related methylation sites. **(B)** The methylation level in TCGA-TNBC subgroups. **(C)** The correlation between expression level of dHRGs and critical genes in m6A process (***, **, and * represent *p* < 0.001, *p* < 0.01, and *p* < 0.05, respectively). The abscissa in **(C)** includes genes involved in the m6A process, Red represents Writers, Blue represents Erasers, and Green represents Readers.

We analyzed the methylation level of dHRGs in TCGA-TNBC patients. We calculated the Spearman rank correlation coefficient between HRGs and methylation site; 14 methylation sites related to expression were obtained (*p* < 0.05) (**Figures 5A, B**), which corresponded to eight genes (**Supplementary Table S6**). The expression levels of these HRGs is negatively correlated with the methylation levels, in which AHNAK2 corresponds to three methylation sites, BHLHE40 corresponds to four methylation sites, and FBXO32 corresponds to two methylation sites. Generally, high methylation level suppressed the expression of gene, which is consistent with our results. RNA N6-methyladinosine (m6A) plays a pivotal role in many biological processes. Therefore, we analyzed the expression of 17 critical genes in the m6A process and performed the correlation analysis between the expressions of eight dHRGs and 17 m6A genes. S100A2, GLRX, and CYB5A are negatively correlated with these 17 genes with low significance. PLOD2, FBXO32, DSC2, BHLHE40, and AHNAK2 showed significantly positive correlation with these 17 genes (**Figure 5C**). According to the regulation process of m6A, the total effect of m6A concerning HRGs on TNBC was promoting tumorigenesis and progression of tumors, due to the favorable prognostic role of the target mRNA, FBXO32, and a positive reader effect (14).

### SCNV and SSNV Characteristics of HRGs

**Figure 6:**
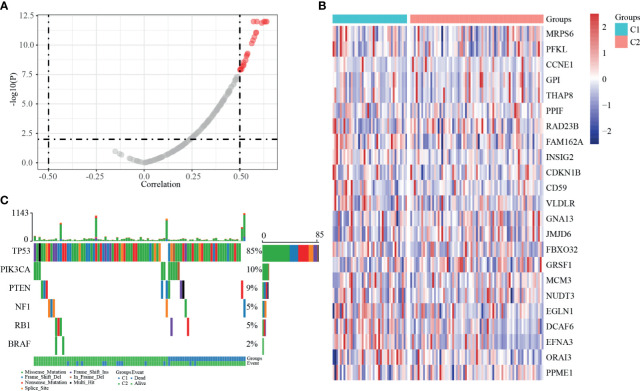
SSNV and SCNV characteristics of dHRGs. **(A)** The correlation coefficient and *p*-value between the expression level of dHRGs and CNV. **(B)** The expression level of 23 dHRGs in different groups of TCGA-TNBC. **(C)** The mutation distribution of specific genes in different groups of TCGA-TNBC.

Consistent with the methods we used above, we evaluated the relationship between expression levels of dHRGS and SCNV. A total of 23 dHRGs showed significantly positive correlation with SCNV (**Figures 6A, B** and **Supplementary Table S7**), which is contrary to the methylation characteristics of dHRGs. Among 23 SCNV-related dHRGS, only one gene (FBXO32) belongs to methylation-related dHRGs, which indicated a mutual exclusive effect between methylation and CNV states. We further evaluated the distribution of mutation states of TP53, RB1, PIK3CA, BRAF, PTEN, and EGFR in Cluster 1 and Cluster 2 (**Figure 6C**) (only 100 samples had mutation data in 115 TCGA-TNBC patients). We used chi-square test to identity the difference between Cluster 1 and Cluster 2 (**Table 3**), showing no difference between Cluster 1 and Cluster 2 (*p* > 0.05).

**Table 3:** Signature gene distribution in a subset of the TCGA-TNBC cohort.

Mutant	C1	C2	*p*
*TP53*			
YES	31	54	1
NO	5	10	
*PIK3CA*			
YES	5	5	0.532
NO	31	59	
*PTEN*			
YES	4	6	1
NO	32	58	
*NF1*			
YES	3	5	1
NO	33	59	
*RB1*			
YES	2	3	1
NO	34	61	
*BRAF*			
YES	0	2	0.7434
NO	36	62	

### Construction and Validation of the Prognostic Model

**Figure 7:**
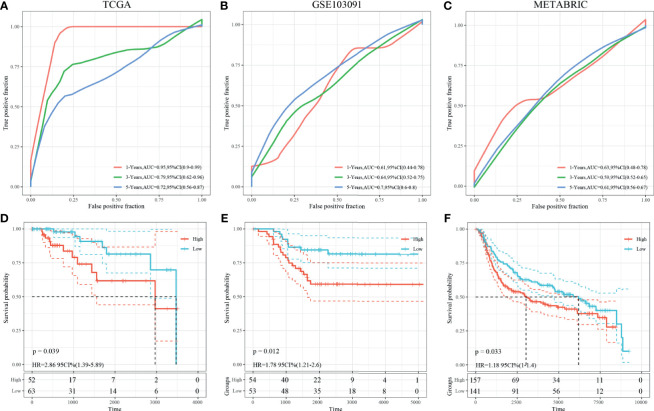
Survival analysis, prognostic performance, and risk score analysis of the HRG-based score model in TNBC patients. The prognostic performance of 3-gene dHRG signature demonstrated by the ROC curve in the TCGA-TNBC cohort **(A)**, GSE103091 cohort **(B)**, and METABRIC cohort **(C)**. K–M survival analysis was performed to estimate the overall survival (OS) of high-risk and low-risk patients in the TCGA-TNBC cohort **(D)**, GSE103091 cohort **(E)**, and METABRIC cohort **(F)**.

Univariate survival analysis was performed on the TCGA-TNBC and GSE103091 cohorts, and we identified three prognosis-associated HRGs. The HRG-based risk score model (defined as 3-gene dHRG signature) was established based on the TCGA-TNBC training set with the following formula: Risk Score = 0.417*ALDOA + 0.569*PFKL + 0.39*PGK1. According to the 3-gene dHRG signature, patients were divided into a high-risk and low-risk group by using the median value of the risk score. K–M survival analysis showed that compared with low-risk patients, high-risk patients had poorer OS in the TCGA-TNBC training set [*p* = 0.039, HR = 2.86, 95 CI% (1.39–5.89)] and GSE103091 [*p* = 0.012, HR = 1.78 95 CI% (1.21–2.6)] (**Figures 7D, E**). ROC analysis demonstrated that the 3-gene dHRG signature performed well in predicting 1-, 3-, and 5-year OS rates, with respective area under the curve (AUC) values of 0.95, 0.79, and 0.72 in the TCGA-TNBC training set (**Figure 7A**). Moreover, the 3-gene dHRG signature showed values in predicting 1-, 3-, and 5-year OS rates, with respective AUC values of 0.61, 0.64, and 0.79 in the GSE103091 cohort (**Figure 7B**). Additionally, the predicting ability of the 3-gene dHRG signature was further verified in the METABRIC dataset in a similar way. A total of 298 patients were divided into high- and low-risk groups *via* using the risk score formula mentioned earlier. K–M survival analysis also indicated that patients with high risk scores in the METABRIC validation set presented a significantly worse OS than those with low risk scores [*p* = 0.033, HR = 1.18 95 CI% (1–1.4)] (**Figure 7F**). ROC analysis also suggested favorable values in predicting OS in the METABRIC dataset (**Figure 7C**).

**Figure 8:**
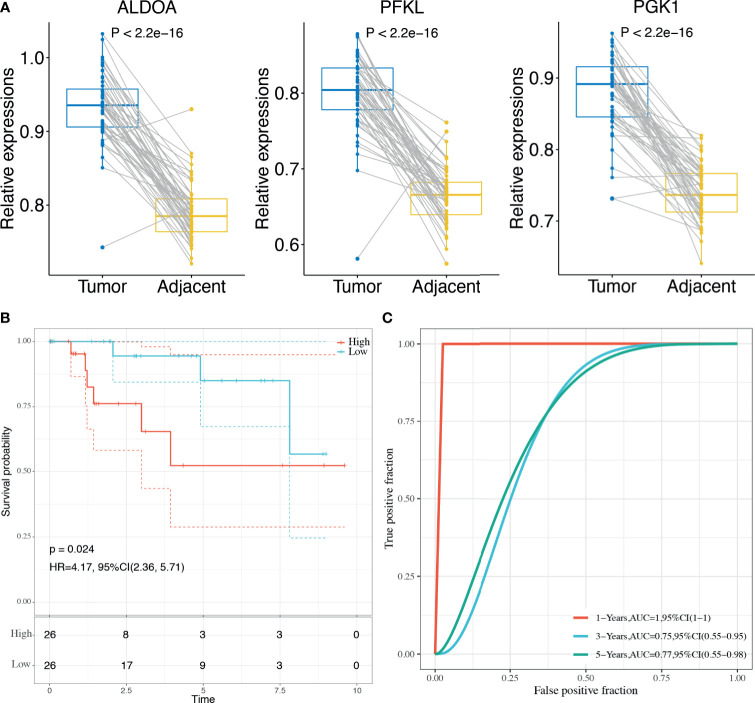
Validation of the HRG-based score model in the BMCHH cohort. **(A)** Comparisons of the expression levels of 3 genes between TNBC tumor samples and paired adjacent normal samples. **(B)** K–M survival analysis was performed to estimate the OS of high-risk and low-risk patients. **(C)** The prognostic performance of 3-gene dHRG signature demonstrated by the ROC curve in the BMCHH validation cohort.

Finally, the 3-gene dHRG signature was also validated in the BMCHH cohort. The expressions of ALDOA, PFKL, and PGK1 were significantly higher in TNBC samples compared with adjacent normal tissues (**Figure 8A**). K–M survival analysis indicated that patients with high risk scores showed significantly poorer OS than those with low risk scores (*p* = 0.024; **Figure 8B**). ROC analysis demonstrated that the 3-gene dHRG signature showed excellent performance in predicting the 1-, 3-, and 5-year OS rates, with respective AUC values of 1.0, 0.75, and 0.77 in the BMCHH validation cohort (**Figure 8C**). All these results suggested that the 3-gene dHRG signature could serve as a robust and reliable prognostic biomarker for OS of TNBC patients from different patient populations.

## Discussion

Hypoxia was a hallmark of TME, which was caused by rapid proliferation of tumor cells and the intercapillary distance longer than that of oxygen diffusion (5). Previous studies have addressed the vital roles of hypoxia status playing in the failure of conventional cancer therapies and poor prognosis of multiple cancer (15, 16). Due to the significant roles of hypoxia, the hypoxia-related gene signatures of glioblastoma (12), colorectal cancer (17), hepatocellular carcinoma (18), bladder cancer (19), renal cell carcinoma (20), and breast cancer (21–24) have been constructed to predict patient survival outcomes. As the most malignant and aggressive breast tumor, TNBC is characterized by severely low tumor oxygenation. Numerous researchers have focused on the association between hypoxia and TNBC. Cox found that lysyl oxidase (LOX) in the hypoxic cancer secretome could disrupt normal bone homeostasis and lead to the formation of focal pre-metastatic lesions in patients with estrogen-receptor-negative breast cancer (25). HRGs and HIFs and their target gene products are known to be hyperactivated in TNBCs, which is proven to be involved in different tumoral mechanisms of TNBC, such as immune evasion, resistance to therapies, invasion, and metastasis (26–28). Therefore, HRGs can be widely used as promising prognostic predictors and therapeutic targets for TNBC. However, there is still a lack of systematic analyses of the prognostic prediction models on the basis of multiple HRGs for TNBC.

In the present study, we first identified hypoxia-related DEGs using the GSE104193 and GSE33950 datasets. By combining with the HRGs, we obtained TNBC-specific hypoxia-related genes, which are defined as dHRGs. Then, we performed univariate analysis and identified 48 dHRGs associated with prognosis, which was defined as PRGs. NMF algorithm was performed using PRGs to classify TNBC patients from TCGA, GSE103091, and METABRIC databases as cluster 1 and cluster 2. These results demonstrated that TNBC patients from different populations can be reliably divided into two clusters on the basis of different hypoxic TME gene patterns. Given the low clinical maneuverability of using 48 genes to predict the survival outcomes of patients, we selected the common PRGs, including PFKL, ALDOA, and PKG1, in both TCGA and GSE103091 cohort to construct the TNBC prognostic risk model (3-gene dHRG signature).

PFKL, ALDOA, and PKG1 are all associated with the glycolytic process. Phosphofructokinase 1 (liver type, PFKL) is glycolytic enzyme, which catalyzes one of the rate-limiting steps of the glycolysis, which has a strong effect on glycolysis (29–31). Aldolase A (ALDOA) is one of glycolytic enzymes mainly found in the developing embryo and adult muscle (32). Gao et al. reported that ALDOA-associated genes plus ALDOA represented a potential new signature for development and prognosis in several cancers (33–35). Phosphoglycerate kinase 1 (PGK1) is the first adenosine triphosphate (ATP)-generating glycolytic enzyme in the aerobic glycolysis pathway (36, 37). As reported in the literature (38, 39), inhibition of PGK1 could suppress aerobic glycolysis by decreasing glucose uptake, lactate and ATP production, extracellular acidification rate, and promoting oxygen consumption rate in breast cancer cells (40). Though there were no specific studies demonstrating the relationship between ALDOA/PFKL/PGK1 and TNBC, a previous study reported that aerobic glycolysis is crucial for modulating breast cancer cell proliferation, invasion, migration, and metastasis both *in vitro* and *in vivo*.

Further analyses demonstrated that the 3-gene dHRG signature could accurately predict the survival outcomes of TNBC patients, which was validated in three independent clinical cohorts (TCGA, GSE03091, and METABRIC). The significant prognostic differences between high-risk and low-risk patients proved the reliability of the 3-gene dHRG signature in predicting prognosis. Hence, our model might be useful tools for assisting both physicians and patients in predicting clinical outcomes and making treatment strategies. According to the 3-gene dHRG model, more active treatment strategies and closer follow-ups should be considered for those TNBC patients with high risk scores.

Besides, these three genes might provide new targets for clinical treatment. Due to the lack of specific target, TNBC is still reliant on chemotherapeutic regimens for systemic treatment, such as cisplatin. However, cisplatin-induced hypoxia bars its long-term efficacy, which promotes the accumulation of hypoxia-inducible factors and cancer stem cell (CSC) enrichment (41). Recent clinical trials have showed efficacy of cisplatin in combinational chemotherapy in comparison to conventional chemotherapeutic approaches for the treatment of TNBC (42–44). Three genes selected in our studies might be an effective target in combinational therapy with cisplatin.

In addition, we also analyzed the methylation characteristics of HRGs, and we found that their expression was negatively correlated with the level of methylation, and the expression of key genes in the m6A process was mainly positively correlated with the HRG expressions. Except for methylation level, there is also SCNV status that affects the expression of dHRGs. SCNV analysis of dHRGs showed that SCNV was positively correlated with their expression, and the overlap rate between methylation-related dHRGs and SCNV-related dHRGs was very low, suggesting that they had a certain mutually exclusive effect on the regulation of HRG expression.

In conclusion, by performing a comprehensive multi-omic analysis based on transcriptomic, DNA methylation, m6A RNA methylation, SCNV, and SSNV patterns, we developed and validated a hypoxic TME-based signature that could be applied for subgrouping and risk stratification for TNBC patients. The prognostic model for OS prediction was further constructed for individualized survival prediction, better treatment decision-making, and follow-up scheduling. However, the present study was limited by the absence of experimental evidence. Further studies concerning *in vitro* and *in vivo* experiments are needed to delineate the molecular mechanism. Besides, large-scale, multicenter, and prospective studies are also needed to verify our prediction model.

## Data Availability Statement

The GSE104193, GSE33950, and GSE103091 datasets analyzed during the current study are available in the GEO repository (http://www.ncbi.nlm.nih.gov/geo/). The RNA sequencing data and clinical information of breast cancer patients were downloaded from The Cancer Genome Atlas (TCGA, http://portal.gdc.cancer.gov/) and METABRIC (http://www.METABRIC.org/) datasets. The qPCR data supporting the conclusions of this article will be made available by the authors, without undue reservation, to any qualified researcher.

## Ethics Statement

The studies involving human participants were reviewed and approved by Research Ethics Committee of Baoji Maternal and Child Health Hospital. The patients/participants provided their written informed consent to participate in this study. Written informed consent was obtained from the individual(s) for the publication of any potentially identifiable images or data included in this article.

## Author Contributions

XS, HL, and CH performed data curation and analysis. XS, YZ, and CY analyzed and interpreted the results. XS and CY drafted and reviewed the manuscript. All authors contributed to the article and approved the submitted version.

## Conflict of Interest

The authors declare that the research was conducted in the absence of any commercial or financial relationships that could be construed as a potential conflict of interest.

## Publisher’s Note

All claims expressed in this article are solely those of the authors and do not necessarily represent those of their affiliated organizations, or those of the publisher, the editors and the reviewers. Any product that may be evaluated in this article, or claim that may be made by its manufacturer, is not guaranteed or endorsed by the publisher.
