# Supplementary figures and images for: Identification of a Hypoxia-Related Molecular Classification and Hypoxic Tumor Microenvironment Signature for Predicting the Prognosis of Patients with Triple-Negative Breast Cancer
Source: Front Oncol. 2021 Aug 19;11:700062. doi: 10.3389/fonc.2021.700062 (PMC8416750; doi:10.3389/fonc.2021.700062)

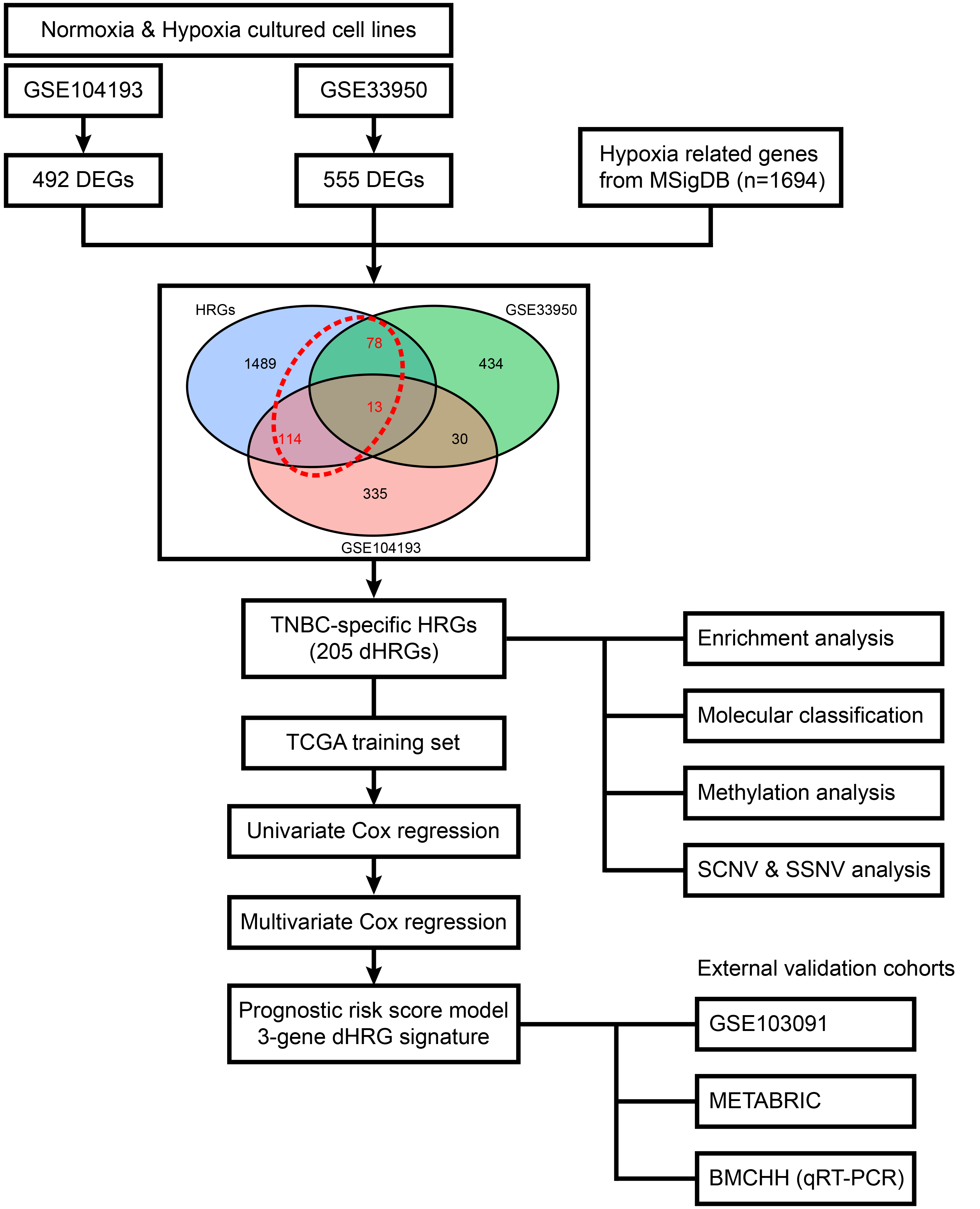

Supplement: Supplementary file 1 [file Image_1.tif]
