# Supplementary material for: Identification of a Hypoxia-Related Molecular Classification and Hypoxic Tumor Microenvironment Signature for Predicting the Prognosis of Patients with Triple-Negative Breast Cancer
Source: Front Oncol. 2021 Aug 19;11:700062. doi: 10.3389/fonc.2021.700062 (PMC8416750; doi:10.3389/fonc.2021.700062)
Supplement: Supplementary file 3 [file Table_2.docx]

**Supplementary Table 2.** Primers for real-time PCR.

| Genes | Forward (5’ to 3’) | Reverse (5’ to 3’) |
| --- | --- | --- |
| ALDOA | ATGCCCTACCAATATCCAGCA | GCTCCCAGTGGACTCATCTG |
| PFKL | GCTGGGCGGCACTATCATT | TCAGGTGCGAGTAGGTCCG |
| PGK1 | TGGACGTTAAAGGGAAGCGG | GCTCATAAGGACTACCGACTTGG |
| GAPDH | TGACTTCAACAGCGACACCCA | CACCCTGTTGCTGTAGCCAAA |
